# Durable Complete Response in a Melanoma Patient With Unknown Primary, Associated With Sequential and Severe Multi-Organ Toxicity After a Single Dose of CTLA-4 Plus PD-1 Blockade: A Case Report

**DOI:** 10.3389/fonc.2020.592609

**Published:** 2020-11-11

**Authors:** Johanna Matull, Elisabeth Livingstone, Axel Wetter, Lisa Zimmer, Anne Zaremba, Harald Lahner, Dirk Schadendorf, Selma Ugurel

**Affiliations:** ^1^ Department of Dermatology, University of Duisburg-Essen, Essen, Germany; ^2^ Department of Diagnostic and Interventional Radiology and Neuroradiology, University of Duisburg-Essen, Essen, Germany; ^3^ Department of Endocrinology, Diabetes and Metabolism and Division of Laboratory Research, University of Duisburg-Essen, Essen, Germany

**Keywords:** checkpoint inhibitors, melanoma, immune-related adverse events, therapy discontinuation, case report

## Abstract

Monoclonal antibodies blocking PD-1 and CTLA-4 immunological checkpoints lead to durable tumor responses in a considerable number of advanced melanoma patients. Besides their anti-neoplastic efficacy, these immune checkpoint inhibitors cause a wide range of immune-related adverse events (irAEs), often enforcing an early discontinuation of therapy. The value of irAEs as a predictive marker for better patient survival is still debated. We report here on a melanoma patient with intramuscular, pulmonary, and bone metastases who developed severe sequential irAEs involving multiple organ systems after single application of a combined immunotherapy with ipilimumab plus nivolumab, followed by a durable complete response despite an early discontinuation of therapy.

## Introduction

Targeting of immune checkpoints has revolutionized the treatment of metastatic melanoma. Anti-PD-1 monotherapy as well as combined PD-1 and CTLA-4 checkpoint inhibition is approved for metastatic melanoma, and response rates of approximately 40% for anti-PD-1 monotherapy and 60% for the combination with anti-CTLA-4, respectively, have been reported in clinical trials (1). The combination checkpoint inhibition of PD-1 plus CTLA-4 modulates the priming as well as the effector phase of the anti-tumoral immune response (2, 3). However, as a result of this strong immunostimulation, a wide range of immune-related adverse events (irAEs) can be induced by this combination therapy, leading to a frequency of 50 to 60% of severe (grades 3 to 4 according to the CTCAE) treatment-related AEs (4). In clinical trials, anti-PD-1 plus anti-CTLA-4 combination immunotherapy had to be stopped during the induction phase within the first 12 weeks in nearly 40% of cases due to severe adverse events. This early discontinuation, however, was not associated with an inferior outcome (3). Early recognition of irAEs and rapid initiation of appropriate treatment measures are important to prevent life-threatening sequelae or death. We here reported a variety of consecutively occurring severe irAEs involving multiple organ systems after a single dose of combined immunotherapy with ipilimumab and nivolumab in a patient with metastatic melanoma, resulting in treatment discontinuation but also in durable complete response (CR).

## Case Presentation

**Figure 1:**
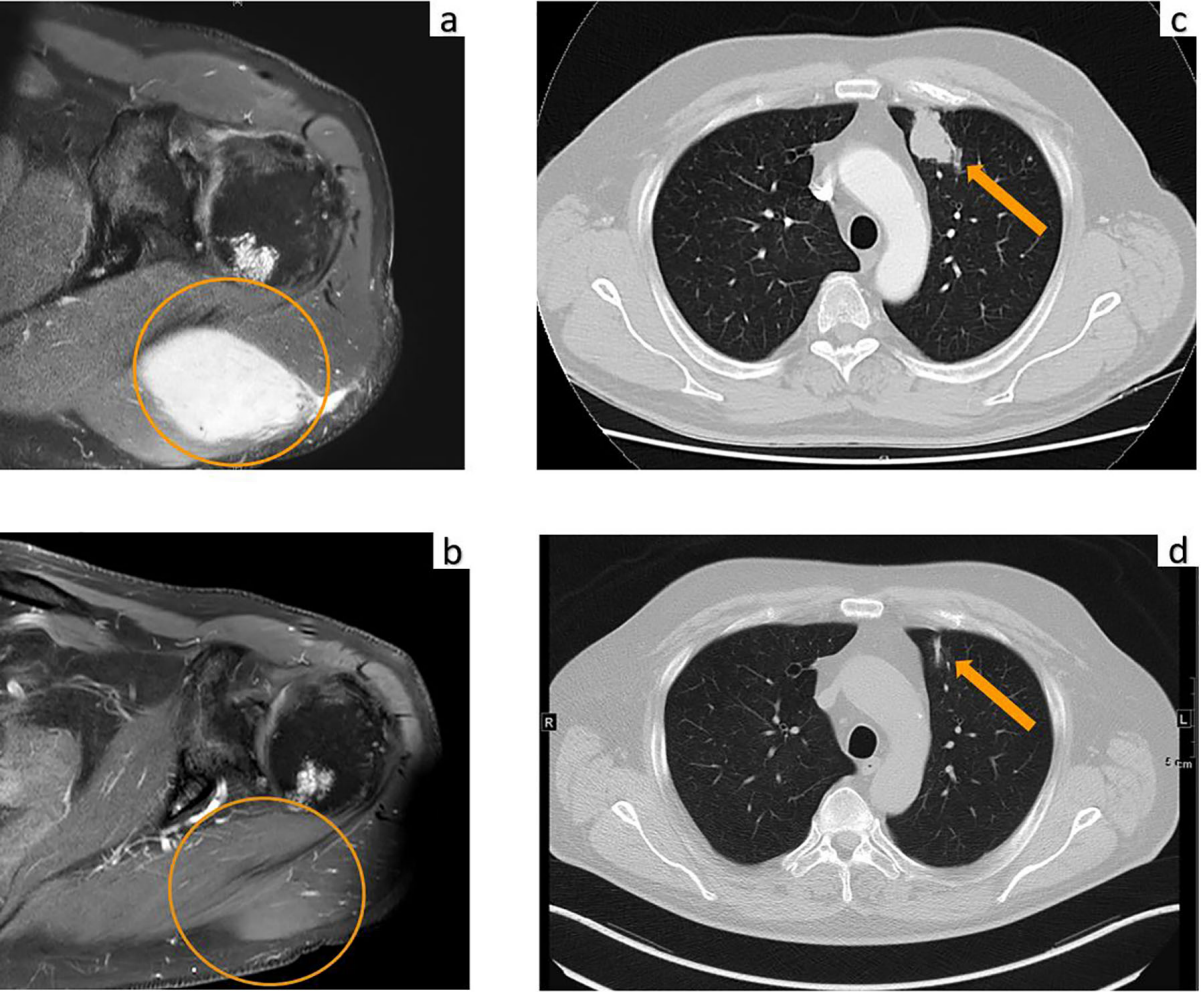
MRI of the upper left extremity: **(A)** Strong contrast enhancement of a 4.0 × 2.6 cm metastasis of the left deltoid muscle before initiation of immunotherapy. **(B)** Complete resolution of the tumor mass at three months after the first and only dose of ipilimumab plus nivolumab. Computed tomography of the chest: **(C)** Metastasis of the left upper lobe (arrow) before initiation of immunotherapy. **(D)** Complete resolution of the lung metastasis with residual dystelectasis (arrow) at three months after the first and only dose of ipilimumab plus nivolumab.

**Figure 2:**
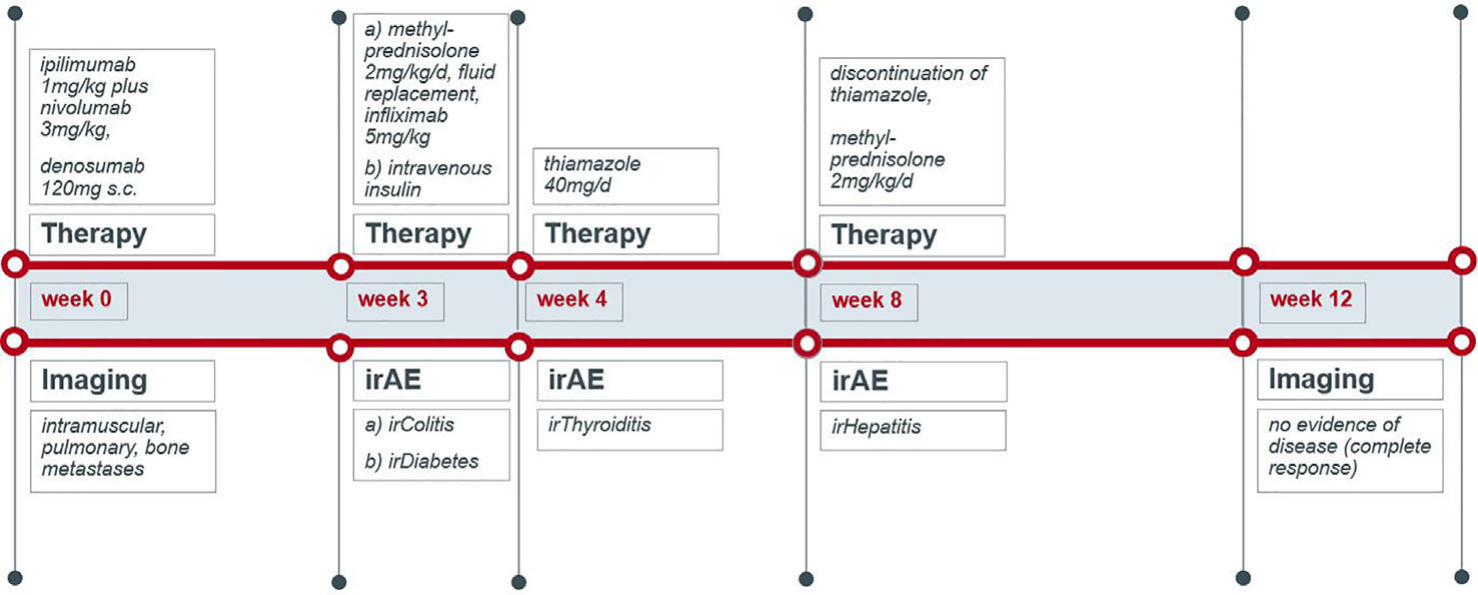
Schematic timeline of immune checkpoint inhibition therapy and its immune-related adverse events.

**Figure 3:**
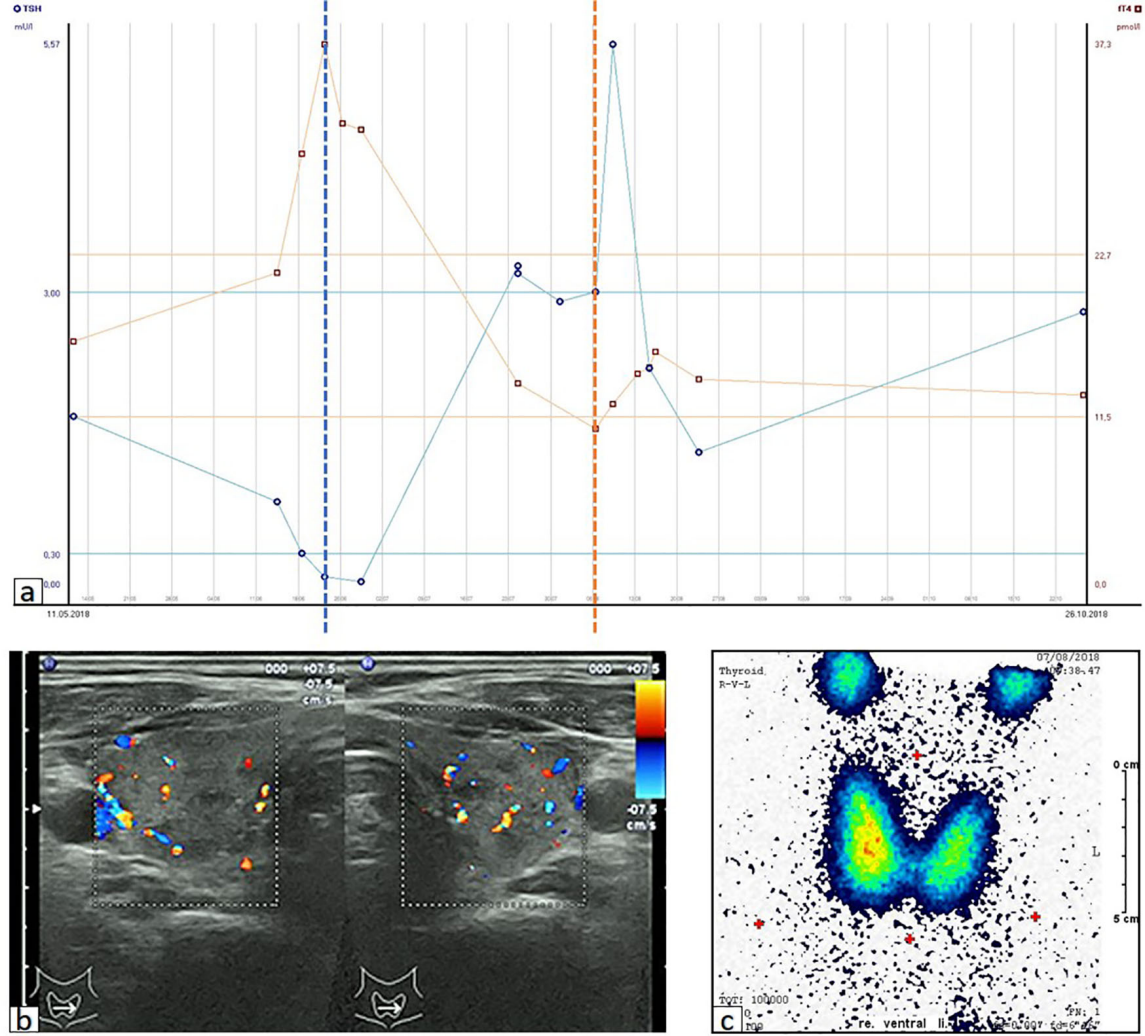
Immune-related thyroiditis. **(A)** Time course of serum parameters reflecting the thyreoiditis after the application of ipilimumab plus nivolumab on 28.05.2018. Dotted lines represent the timepoint of initiation/discontinuation of thiamazole. TSH, thyroid stimulating hormone; fT4, free thyroxine; LLN, lower limit of normal; ULN, upper limit of normal. **(B)** Ultrasound showing hypoechoic, heterogeneous and hypervascular thyroid gland. **(C)** Tc99m scintigraphy showing proportional, slightly elevated tracer uptake (2.6%) throughout the thyroid gland.

A 70-year-old male was diagnosed with metastatic melanoma in March 2018 after presenting with a painless subcutaneous swelling of the left scapular region that had increased noticeably in size over the last few months. MRI scans revealed a tumor lesion of 4 cm in diameter within the left deltoid muscle (**Figure 1A**). An incisional biopsy confirmed the diagnosis of melanoma. Subsequent CT and FDG-PET scans detected multiple intramuscular, pulmonary, and bone lesions (**Figure 1C**). The primary tumor could not be detected even after a thorough physical examination so that the diagnosis of a melanoma of unknown primary was made. Serum LDH and S100B levels were within normal range. Molecular analysis of the tumor tissue biopsy revealed a mutation of the *NRAS* gene (Q61L); *BRAF*, *KIT*, and *NF1* genes were wildtype, and the PD-L1 surface expression on tumor cells was negative (0%). After interdisciplinary tumor board discussion, a combined immunotherapy with ipilimumab (3 mg/kg) plus nivolumab (1 mg/kg) Q3W was initiated (for timelines see **Figure 2**). As concomitant therapy of bone lesions, the patient received the RANK-L inhibitor denosumab (120 mg Q4W). Sixteen days after the first application of ipilimumab and nivolumab, the patient presented with a sudden onset of severe diarrhea with >10 stools/d, weight loss of 3 kg, exsiccosis, and severe deterioration of his general condition. An immune-related colitis (CTCAE grade 4) was diagnosed, and the patient was hospitalized for intravenous administration of methylprednisolone (2 mg/kg/d) and fluid replacement. After 48 h with no improvement of symptoms, the TNF alpha inhibitor infliximab (5 mg/kg) was added, which led to a complete recovery from diarrhea within 7 days. In addition, a significant elevation of serum amylase and lipase levels (CTCAE grade 4) was noted on the day of admission. One day later, blood tests demonstrated the sudden onset of severe hyperglycemia (glucose 33 mmol/L) and ketoacidosis with inadequately low insulin and c-peptide levels. The patient was diagnosed with an immune-related diabetes mellitus (CTCAE grade 4) and after transferral to the intermediate care unit was carefully substituted intravenously with insulin. Three days later, the patient was switched to subcutaneous insulin application. Four weeks after the first and only application of ipilimumab and nivolumab, the patient developed manifest hyperthyroidism (TSH 0.02 mU/L, normal range 0.3–3.0 mU/L; fT4 37.3 pmol/L, normal range 11.5–22.7 pmol/L) with negative TSH receptor antibodies; **Figure 3A**. Ultrasound showed a hypoechoic, heterogeneous, and hypervascular thyroid gland, and Tc99m thyroid scintigraphic scan revealed a slightly elevated uptake in both thyroid lobes corresponding to autoimmune thyroiditis; **Figures 3B, C**. The patient was started on thyrostatic therapy with thiamazole 40 mg/d leading to euthyroidism after 8 weeks. Corticosteroids were tapered slowly and the patient was discharged after 5 weeks of hospitalization in a good general condition. Another four weeks after discharge, laboratory tests revealed a rapid and strong increase in serum levels of alanine transaminase (ALT) and aspartate transaminase (AST) CTCAE grade 4. The patient was diagnosed with an immune-related hepatitis; however, a toxic hepatopathy induced by thiamazole could not be ruled out. The patient was again hospitalized, thiamazole was discontinued, and high-dose intravenous methylprednisolone (2 mg/kg/d) was re-initiated. Upon corticosteroids, the liver enzyme levels rapidly normalized, and the patient could be discharged after another 14 days. Three months after the first and only dose of ipilimumab plus nivolumab, CT and MRI scans showed no evidence of disease (**Figures 1B, D**), and the patient was classified as a complete responder. At the same time, all therapy-related side effects had completely resolved, all laboratory parameters had returned to normal values, and the patient did not need further substitution therapy of insulin and thyroxin. The latest F18FDG PET/CT was performed on June 18, 2019 without any metabolic abnormality. S100B levels in the peripheral blood were still within normal range (0.05 µg/L) in January 2020. Subsequently, the patient was lost to follow-up.

## Discussion

Checkpoint inhibitors such as PD-1 and CTLA-4 antibodies can cause a wide range of irAEs. The types of irAEs and the affected organs are similar for all checkpoint inhibitors, but their frequency and grade of severity are higher for the combination treatment with anti-CTLA-4 plus anti-PD-1 than for the respective monotherapies (5, 6). The randomized phase-3 trial CheckMate-067 showed that hepatitis, colitis, as well as thyroiditis are common side effects of combined checkpoint blockade. Hepatitis and/or elevated liver enzymes were observed in up to one-third of patients treated with ipilimumab plus nivolumab, followed by colitis (13%) and hyperthyroidism (11%) (7). In contrast, immune-related pancreatitis with diabetes mellitus belongs to the rare side effects of checkpoint inhibitor therapy (8, 9). The present case is remarkable for the consecutive occurrence of numerous severe irAEs affecting various organ systems after single administration of ipilimumab plus nivolumab, but also for the complete resolution of all irAEs. irAEs in more than one organ system were reported in 25% of patients treated with nivolumab plus ipilimumab (10); the percentage of patients developing three or more irAEs has not been characterized precisely. In addition, the rapid onset of the individual irAEs observed in our case is unusual. A pooled analysis of patients treated with ipilimumab (10 mg/kg Q3W) showed a characteristic pattern in the timing of the occurrence of distinct irAEs (11). Gastrointestinal irAEs usually arise six to seven weeks after therapy start. In our case, colitis and pancreatitis occurred only three weeks after the first application of immunotherapy. Endocrinal side effects can be expected at approximately nine weeks after treatment start. In our patient, immune-related diabetes was observed at three weeks, and immune-related hyperthyroidism at four weeks after therapy started. It is also remarkable that our patient achieved a deep and durable complete response after only one administration of combined immunotherapy. Repeated imaging only three months after treatment started showed no evidence of disease.

Despite high response rates and durable response—as in the present case—a subset of patients does not respond to immunotherapy or initially respond and finally relapse. Research efforts are underway to identify new agents that could be targeted in combination with immune checkpoint inhibitors to achieve synergistic anti-tumor effects. In the case reported here, the patient received supportive therapies, including infliximab and denosumab, which could have influenced the anti-tumor response. While denosumab with its osteoclastogenetic effect is used to prevent skeletal related events in patients with bone metastases, infliximab is administered for steroid-refractory immune mediated toxicity. There is growing evidence that both infliximab and denosumab can enhance the effectiveness of immunotherapy. In a retrospective observational study combination of denosumab with immune checkpoint inhibition improved overall survival compared with monotherapy in metastatic melanoma patients (12). Likewise, in a case series with 29 melanoma patients, the combination of PD-1 inhibition with denosumab showed a promising efficacy without an increase in toxicity (13). It is assumed that the RANK-RANKL signaling pathway plays a role in tumorigenesis and that denosumab exerts a direct anti-tumor effect by inhibiting this pathway (14). The combination of immunotherapy and Infliximab does not seem to negatively impact anti-tumor efficacy either (15). Rather, preclinical studies have shown that TNF blockade can increase the therapeutic response to anti-PD-1 (16). It is conceivable that both denosumab and infliximab have increased the effectiveness of ipilimumab and nivolumab in our reported case.

Discontinuation of checkpoint inhibition due to irAE was not associated with a worse treatment outcome in our study. Indeed, there is evidence of a possible association of irAEs with a favorable therapy outcome. In a retrospective analysis, Weber et al. showed that in 576 patients treated with nivolumab monotherapy (excluding those who had progressed prior to week 12), there was no difference in progression-free survival (PFS) between patients without AEs. Similar observations were made for patients with one to two AEs or between those with any-grade AE and all patients (17). In a retrospective analysis of irAEs in patients treated with nivolumab plus peptide vaccine or nivolumab alone, Freeman-Keller et al. noted an overall survival (OS) benefit in patients showing a rash and a vitiligo; no significant survival benefit was seen with other irAEs including endocrinopathies, colitis, and pneumonitis (18). Swami et al. reviewed single institution data of 169 patients treated with anti-PD1-therapy of which 16 discontinued treatment due to irAE and observed a durable clinical benefit in 13 (81.2%) patients (19). Another study by Quach et al. reported cutaneous side effects to be correlated with a favorable response rate and median OS in patients treated with anti-PD-1 therapies. Superior outcomes regarding response rate, PFS, and OS were seen in patients showing a vitiligo and rash as compared to those presenting with pruritus (20). Indini et al. reported an independent association of irAEs with improved PFS and OS on multivariate analysis in patients treated with nivolumab or pembrolizumab (21). Maher et al. observed an association between AEs and tumor response in an examination of seven trials in 1,747 patients with metastatic or locally advanced urothelial cancer treated with anti-program death protein 1 or ligand 1 antibodies. In these analyses a related AESI was reported in 64% of responding patients and in 34% of patients who did not respond to the anti-PD-1/PD-L1 antibody (22). In our patient, the occurrence of multiple and severe irAEs was associated with an extremely favorable therapy outcome of ipilimumab plus nivolumab, reflected by a deep response of CR at three months after therapy onset durably lasting for 19 months.

Given the methodological limitations of our findings being a case report, the value of irAEs as a predictive marker for better patient survival and response to treatment with immune checkpoint inhibition requires confirmation by further studies on large patient cohorts. In addition, having the patient an unknown primary melanoma, the history of the disease and its clinical progression rate before therapy cannot be evaluated, thus limiting the evaluation of the efficacy of a single course of treatment in reaching a complete response.

## Data Availability Statement

The original contributions presented in the study are included in the article/supplementary material. Further inquiries can be directed to the corresponding author.

## Ethics Statement

Written informed consent was obtained from the individual(s) for the publication of any potentially identifiable images or data included in this article.

## Author Contributions

All authors (JM, EL, AW, LZ, AZ, HL, DS, and SU) contributed to data collection, critical revision of the paper, and final approval of the version to be published. JM and SU conceived the work and wrote the paper. All authors contributed to the article and approved the submitted version.

## Conflict of Interest

JM declares travel support from Bristol Myers Squibb, Novartis and Sun Pharmaceutical Industries. EL declares consulting for Actelion, Bristol-Myers Squibb, MSD, and Novartis; advisory role for Roche, Bristol-Myers Squibb, MSD, and Novartis; honoraria from Amgen, Bristol-Myers Squibb, MSD, Novartis, Janssen, and Medac; travel grants from Medac, Novartis, Bristol-Myers Squibb, Amgen, Pierre Fabre, and Sun Pharma. LZ has served as consultant and/or has received honoraria from Roche, Bristol Myers Squibb, Merck Sharp and Dohme, Novartis, Pierre Fabre, and Sanofi, and received travel support from Bristol Myers Squibb, Merck Sharp and Dohme, Amgen, Pierre Fabre and Novartis. AZ received travel support from Novartis and Bristol-Myers Squibb. DS declares advisory board and speakers honoraria from Roche, Novartis, Bristol-Myers-Squibb, MSD, Merck-Serono, Sanofi, Nektar, Amgen, Hexal, InFlaRx, Array, Pierre Fabre, Immunocore, Philogen Sun Pharma, Regeneron and Ultimovacs as well as grant and travel support from Roche, Novartis, Bristol-Myers-Squibb, MSD, Merck-Serono, and Sanofi. SU declares research support from Bristol Myers Squibb and Merck Serono; speakers and advisory board honoraria from Bristol Myers Squibb, Merck Sharp & Dohme, Merck Serono, Novartis and Roche, and travel support from Bristol Myers Squibb, and Merck Sharp & Dohme.

The remaining authors declare that the research was conducted in the absence of any commercial or financial relationships that could be construed as a potential conflict of interest.
